# PG-2, a Potent AMP against Pathogenic Microbial Strains, from Potato (*Solanum tuberosum* L cv. Gogu Valley) Tubers Not Cytotoxic against Human Cells

**DOI:** 10.3390/ijms14024349

**Published:** 2013-02-21

**Authors:** Jin-Young Kim, Ramamourthy Gopal, Sang Young Kim, Chang Ho Seo, Hyang Burm Lee, Hyeonsook Cheong, Yoonkyung Park

**Affiliations:** 1 Research Center for Proteineous Materials, Chosun University, Gwangju 501-759, Korea; E-Mails: jyfrog@hanmail.net (J.-Y.K.); ramagopa@gmail.com (R.G.); 2 Department of Bioinformatics, Kongju National University, Kongju 314-701, Korea; E-Mails: sinji1108@gmail.com (S.Y.K.); chseo@kongju.ac.kr (C.H.S.); 3 College of Agriculture and Life Sciences, Chonnam National University, Gwangju 501-759, Korea; E-Mail: hblee@jnu.ac.kr; 4 Department of Biotechnology, Chosun University, Gwangju 501-759, Korea; E-Mail: hscheong@chosun.ac.kr

**Keywords:** peptide-G2, RP-HPLC, *N*-terminal sequence, human pathogenic, antimicrobial agent

## Abstract

In an earlier study, we isolated potamin-1 (PT-1), a 5.6-kDa trypsin-chymotrypsin protease inhibitor, from the tubers of a potato strain (*Solanum tuberosum* L cv. Gogu Valley). We established that PT-1 strongly inhibits pathogenic microbial strains, but not human bacterial strains, and that its sequence shows 62% homology with a serine protease inhibitor. In the present study, we isolated an antifungal and antibacterial peptide with no cytotoxicity from tubers of the same potato strain. The peptide (peptide-G2, PG-2) was isolated using salt-extraction, ultrafiltration and reverse-phase high performance liquid chromatography (RP-HPLC). Matrix-assisted laser desorption/ionization time-of-flight mass spectrometry (MALDI-TOF/MS) showed the protein to have a molecular mass of 3228.5 Da, while automated Edman degradation showed the *N*-terminal sequence of PG-2 to be LVKDNPLDISPKQVQALCTDLVIRCMCCC-. PG-2 exhibited antimicrobial activity against *Candida albicans*, a human pathogenic yeast strain, and *Clavibacter michiganensis* subsp. *michiganensis*, a plant late blight strain. PG-2 also showed antibacterial activity against *Staphylococcus aureus*, but did not lyse human red blood cells and was thermostable. Overall, these results suggest PG-2 may be a good candidate to serve as a natural antimicrobial agent, agricultural pesticide and/or food additive.

## 1. Introduction

Within plants and animals, antimicrobial peptides (AMPs) are usually found on surfaces most likely to come into contact with pathogens from the surrounding environment, especially epithelial surfaces and plant cell walls. They are also produced by phagocytes and appear to be involved in bacterial cell killing [1–7]. Indeed, AMPs have been detected in a wide variety of agricultural plant species, where they have been implicated in mediating plant resistance to microbial infection. The presence of AMPs in numerous plant types, along with their potent antimicrobial activity *in vitro*, suggests AMPs play a broadly protective role against plant pathogens. Consistent with that idea, AMPs are strongly expressed both locally and systemically during pathogen attacks [8].

Several AMPs have been purified from potato tubers. For example, the 5-kDa peptide pseudothionin from *Solanum tuberosum* (Pth-St1) is active against *Clavibacter michiganensis* subsp. *sepedonicus*, a bacterial pathogen affecting potatoes, as well as *Pseudomonas solanacearum* and *Fusarium solani*, two other potato pathogens [9]. In addition, snakin-1 and snakin-2 were found to be active against *C. michiganensis* subsp*. sepedonicus*, as well as *Botrytis cinerea*, a fungal pathogen affecting potatoes and other plant species [10,11]. Peptide-G is a small (5578.9 Da) AMP isolated from potato tubers (*Solanum tuberosum* L cv. Golden Valley) that potently inhibits the growth of a variety of bacterial (*Staphylococcus aureus*, *Listeria monocytogenes*, *Escherichia coli* and *C. michiganensis* subsp*. michiganensis*) and fungal (*Candida albicans* and *Rhizoctonia solani*) strains [12]. In an earlier study, we reported the isolation of a trypsin-chymotrypsin protease inhibitor, PT-1, from potato tubers (*Solanum tuberosum* L cv. Gogu Valley) [13]. In the present study, we purified a potent AMP (peptide-G2, PG-2) from the same potato strain and showed that PG-2 exhibits potent inhibitory activity against human and plant pathogens, including *S. aureus*, *C. michiganensis* subsp*. michiganensis* and *C. albicans.*

## 2. Results and Discussion

### 2.1. Purification of PG-2 from Potato Tubers

**Figure 1:**
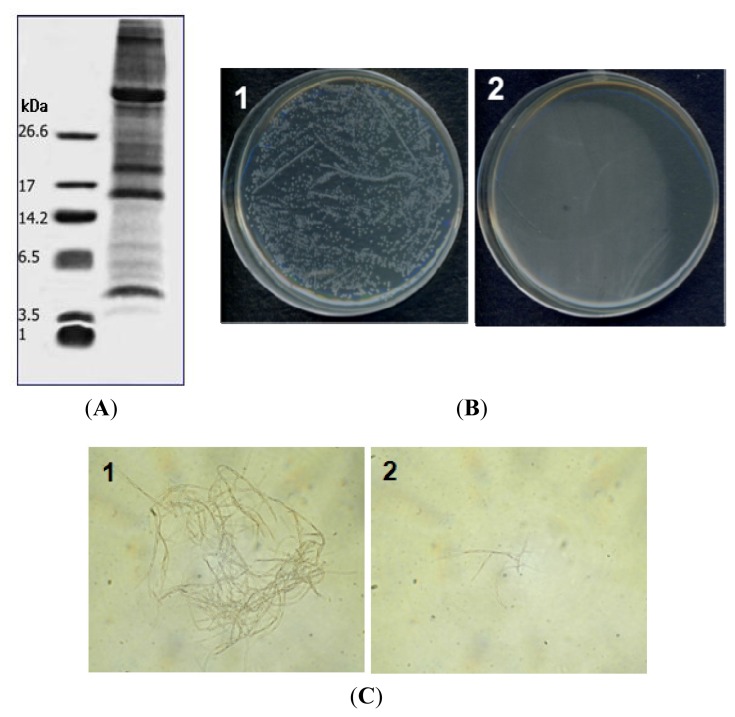
(**A**) Tricine gel electrophoresis of proteins extracted from potatoes. Total proteins were extracted from potato tubers using extraction buffer (10 mM Tris-HCl, 1.5 M LiCl, pH 7.4): lane 1, molecular size markers (26.6, triosephosphate isomerase; 17, myoglobin; 14.2, α-lactalbumin; 6.5, aprotinin; 3.5, insulin chain B; 1, bradykinin); lane 2, proteins extracted from potato (20 μg); (**B**) Antibacterial activities of proteins extracted from potato against the bacterial strain *Clavibacter michiganensis* subsp*. michiganensis*: 1, untreated cells; 2, cell treated with 100 μg of protein (<10 K fraction); (**C**) Antifungal activity against *Rhizoctonia solani*: 1, untreated cells; 2, cells treated with 200 μg of protein (<10 K fraction). Measurements were made in triplicate.

The antimicrobial peptide PG-2 was purified from potato tubers (*Solanum tuberosum* L cv. Gogu Valley) using a four-step protocol: extraction, heating, ultrafiltration and C_18_ reverse-phase HPLC. Total proteins were extracted from potato tubers using extraction buffer (Figure 1A) and then dialyzed against ddH_2_O. The resultant suspension was heated at 70 °C for 20 min to obtain heat-stable peptides, while removing high-molecular weight proteins. The heat-stable peptides were then selected for size by ultrafiltration (10 K) and the <10 K fraction was found to be active against *C. michiganensis* subsp*. michiganensis* (Figure 1B) and *R. solani* (Figure 1C). We subjected the active fraction to C_18_ RP-HPLC, and the antimicrobial activity was collected using the following stepwise gradients: 10%–20%, 20%–30%, 30%–40%, 40%–50% and 50%–95%. The 30%–40% fraction showed antimicrobial activity and was therefore selected for further purification.

**Figure 2:**
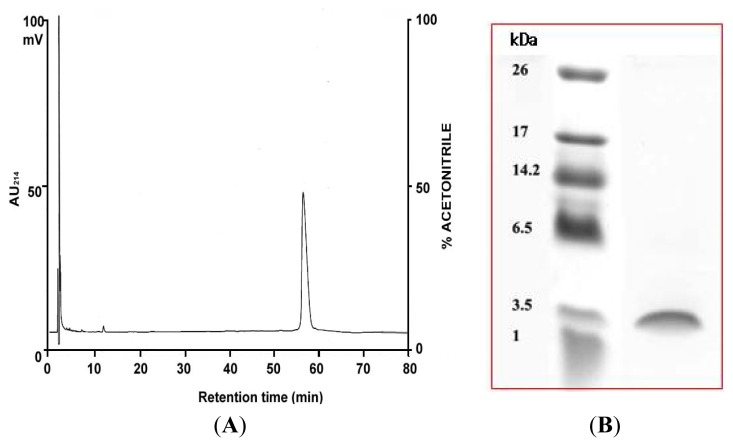
High-performance liquid chromatography (HPLC) profile obtained by re-analyzing peak 5. The protein was eluted using a linear gradient of acetonitrile in 0.1% trifluoroacetic acid (TFA). Absorbance was monitored at 214 nm. (**A**) Peptide-G2 (PG-2) peaks; (**B**) Tricine gel electrophoresis of purified PG-2: lane 1, molecular size markers (same as in Figure 1); lane 2, purified PG-2 (10 μg).

The HPLC program was modified to include a linear gradient (data not shown), and the previously freeze-dried active fraction was further purified by re-running it using a delayed gradient at the time point of the peak (Figure 2A). Of the five peak fractions collected, only peak 5 was active against pathogens in antimicrobial assays. Subsequent 16.5% tricine sodium dodecyl sulfate polyacrylamide gel electrophoresis (SDS-PAGE) showed peak 5 to contain a single molecule with a low molecular weight of around 3300 Da (Figure 2B). We named the molecule peptide-G2 (PG-2).

As summarized in Table 1, the yield of PG-2 peptide from potato tuber was 0.12 mg of pure PG-2 per 200 g of potato tuber.

### 2.2. Antimicrobial Activity

**Figure 3:**
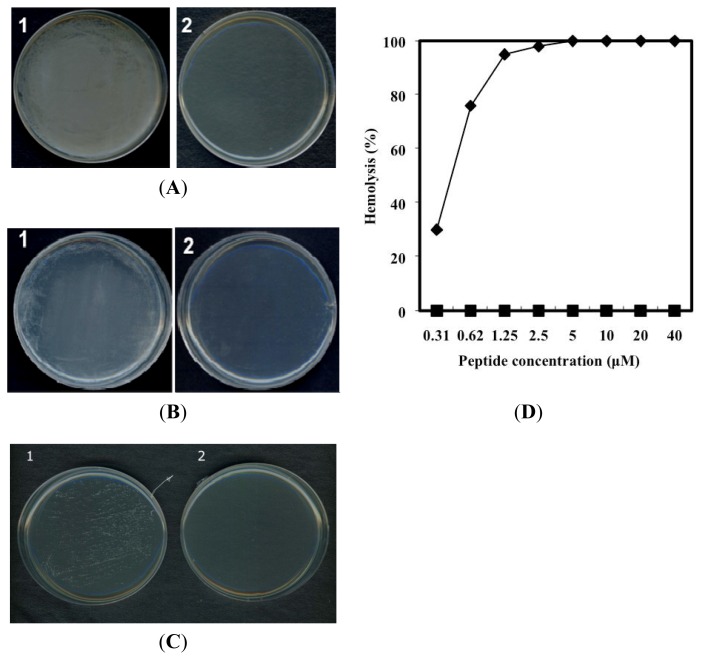
Assays of PG-2 activity against bacterial and fungal strains. (**A**) Antibacterial activity of PG-2 against the plant pathogen *Clavibacter michiganensis* subsp*. michiganensis*: 1, untreated; 2, PG-2 (6.25 μM); (**B**) Antifungal activity of PG-2 against the human pathogen *Candida albicans*: 1, untreated; 2, PG-2 (50 μM); (**C**) Antibacterial assay of PG-2 against the human pathogen *Staphylococcus aureus*: 1, untreated; 2, PG-2 (6.25 μM); (**D**) Hemolytic activities of PG-2 and melittin. Comparison of PG-2- and melittin-induced lysis of human erythrocytes: melittin (◆), PG-2 (▢).

The antimicrobial activities of PG-2 were tested against *C. albicans* and *S. aureus*, a pathogenic yeast and a bacterial strain, respectively, as well as *C. michiganensis* subsp*. michiganensis* and *R. solani*, two plant late blight strains (Table 2). The minimal inhibitory concentration (MIC) for PG-2 was 25 μM against *C. albicans* and *R. solani* and 3.12 μM against *C. michiganensis* subsp*. michiganensis* and *S. aureus* (Figure 3A–C).

### 2.3. Hemolytic Activity of PG-2

For a peptide to be useful as an antibiotic agent, its cytotoxicity must be low. We therefore assessed the hemolysis induced by PG-2 against hRBCs. The hemolytic activity of PG-2 was determined to be 0% at concentrations up to 40 μM, whereas melittin, a positive control, induced about 100% hemolysis at 2.5 μM (Figure 3D). Thus, PG-2 appears to exhibit remarkable antimicrobial activity against a variety of microbial cells, but to have no hemolytic activity.

### 2.4. *N*-Terminal Amino Acid Sequence Analysis of PG-2

**Figure 4:**
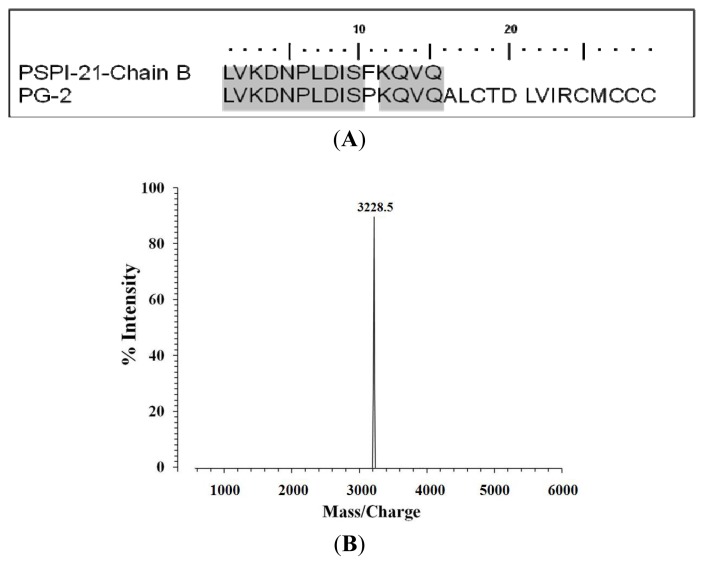
(**A**) Sequence of PG-2 isolated from potato tubers. The *N*-terminal 29 amino acids were sequenced using the Edman degradation method; (**B**) Determination of the molecular mass of PG-2 using matrix-assisted laser desorption/ionization mass spectrometry (MALDI-MS).

The *N*-terminal amino acid sequence of PG-2 was analyzed using Edman degradation with a pulse liquid automatic sequencer (Applied Biosystems Inc., Foster city, CA, USA, model 473A) at the Korea Basic Science Institute (Seoul, Korea). In a BLAST2 peptide sequence homology search of the NCBI database, PG-2 was found to be highly similar to the *C*-terminal portion of protease inhibitors; the best score (43%) was obtained for the serine protease inhibitor 1 precursor (PSPI-21-Chain B) (Figure 4A). In addition, MALDI-TOF/MS analysis showed the peptide’s molecular weight to be 3228.5 Da (Figure 4B).

### 2.5. AMPs from Potatoes

Potatoes are the most important tuber crop in the world in terms of production, accounting for about 45% of the total world production of all tuber crops. In Korea, potatoes are traditionally a main source of carbohydrates and dietary protein, and potato slices or kneaded potato powder is applied to minor burns as a folk remedy.

In an earlier report, antibiotic peptides/proteins purified from potato tubers were divided into three classes. The first class includes the major protein in potato tubers, the globulin tuberin. It also includes a 45-kDa glycoprotein, patatin, which accounts for about 20% of the total soluble protein content of potatoes. Patatin exhibits acyl hydrolase activity, acting as a phospholipase with phospholipid and lysophospholipid substrates, as well as esterase activity. In one recent study, patatin was also shown to act as an acidic β-1,3-glucanase, thereby contributing to plant defense against fungal pathogens through digestion of β-1,3-glucans in hyphal cell walls. In addition, β-1,3-glucanase also serves as a component of the pathogenesis-related (PR) protein response [14].

The second class of proteins is the potato antimicrobial peptide, which include Pthe-St1, snakin-1 and snakin-2. These proteins were isolated from an insoluble extraction of potato tubers and possess potent antimicrobial activities. The cell wall-associated peptide snakin-1 was initially purified as a member of what appears to be a widely distributed peptide type, the snakin/GASA family. Snakin-1 accumulates in different tissues of potato plants during development, and the expression of its gene is unaffected by a variety of abiotic and biotic challenges. Snakin-2, which represents a widely divergent snakin subfamily, is also active *in vitro* against bacterial and fungal plant pathogens, but in contrast to snakin-1, expression of its gene is affected by wounding and pathogens. Notably, although snakin/GASA peptides share only 38% sequence similarity, they are all basic, rich in Cys residues and have almost identical antibacterial activity spectra [11].

The third class of potato-derived AMPs are protease inhibitors and are ubiquitously abundant in tubers and plant seeds [15]. Protease inhibitors in plants are generally thought to function as storage proteins and to constitute a part of a plant’s defense system [16]. For example, these proteins contribute to the wound-induced defense response elicited in plant by wounding and pathogens [17]. They also modulate in wild plant in response to wounding- and herbivory-induced responses [18]. In recent years, there has been renewed interest in protease inhibitor, Bowman-Birk inhibitors (BBI), which have latent health-promoting properties contained by the mammalian gastrointestinal tract [19].

The goal of our work was to isolate and purify a low-molecular weight potato peptide with antimicrobial activity. At present, the development of plant defense systems and novel ecofriendly strategies for the protection of plants against pests and pathogens is one of the most dynamic areas of plant research. Numerous studies have shown that a wide variety of proteins and peptides are involved in defending potato tubers, and the results obtained in the present study suggest that PG-2 may be crucially involved in the defense of its host plant against phytopathogens. Furthermore, its lack of cytotoxic effects against hRBCs suggests PG-2 could potentially serve as a clinically effective antimicrobial agent.

## 3. Experimental Section

### 3.1. Materials

Potato tubers (*Solanum tuberosum* L cv. Gogu Valley) were obtained from Potato Valley at Kang-won National University. Molecular weight marker proteins were purchased from Sigma. The reverse-phase column used for purification of PG-2 was a Vydac C_18_. Acetonitrile and water (HPLC grade) were obtained from Burdick & Jackson Inc. (Muskegon, MI, USA) and trifluoroacetic acid (TFA) was from Merck. Other chemicals and reagents were all of analytical grade.

### 3.2. Bacterial and Fungal Strains for Antimicrobial Activity

The *C. michiganensis* subsp*. michiganensis* (KCTC 9231), *S. aureus* (KCTC 1621) and *C. albicans* (KCTC 7270) strains used in this study were obtained from the Korea Collection for Type Cultures (KCTC) at the Korea Research Institute of Bioscience and Biotechnology (KRIBB, in Daejon, Korea); *R. solani* (KACC 40138) was obtained from the Korea Agricultural Culture Collection (KACC in Suwon, Korea).

### 3.3. Purification of PG-2

Step 1: Extraction and isolation using ultrafiltration. PG-2 was isolated using a previously published method [9]. Potato tuber material (300 g) was first ground to powder in liquid nitrogen using a mortar and pestle. This was followed by extraction in 600 mL of buffer (100 mM Tris-HCl, 1.5 M LiCl, pH 7.2) for 3 h at 4 °C, followed by centrifugation at 12,000 rpm for 20 min. The resultant supernatant was dialyzed against ddH_2_O using a 1000 Da molecular weight membrane, after which the dialysate was heated at 70 °C for 20 min to obtain heat-stable peptides. The heat-denatured precipitates were removed by centrifugation at 24,000 rpm for 30 min. The proteins in the supernatant were then selected for size by ultrafiltration, with cut-offs at 30 and 10 kDa, and freeze-dried.

Step 2: Purification of PG-2 by C_18_ HPLC. The PG-2-containing extract was applied to a C_18_ reverse phase column (Vydac, 4.6 × 250 mm) on an HPLC system (Shimadzu, Japan). The peptide was dissolved in 0.1% (v/v) TFA in HPLC grade water (Solvent A) and loaded onto a C_18_ RP-HPLC column in equilibrium with 0.1% TFA. The peptide was eluted using the following step gradient (Solvent B is acetonitrile containing 0.1% TFA): 0–25 min, 15% solvent B; 25–85 min, 30% solvent B; 85–90 min, 30% solvent B; 90–110 min, 40% solvent B; 110–120 min, 40% solvent B. The eluate was monitored by measuring the absorbance at 214 nm. Each fraction was pooled and freeze dried, after which peptide-containing fractions were re-run on the C_18_ HPLC using a delayed gradient at the peak time point. The peak-containing fraction was then collected and assayed for antimicrobial activity. To confirm the purified peptide was a single molecule, tricine SDS-PAGE and mass spectrum analysis were carried out.

### 3.4. Tricine Sodium Dodecyl Sulfate-Polyacrylamide Gel Electrophoresis

A 16.5% tricine acrylamide gel, based on the formulation of Schagger and von Jagow [20], was used to resolve the peptide isolated by HPLC. Samples were diluted to 0.5 mg/mL in sample buffer containing 0.5% (*w*/*v*) SDS, 50 mM Tris-HCl, pH 6.8, 12% (*v*/*v*) glycerol, 2% 2-mercaptoethanol and 0.01% Brilliant Blue G, after which samples containing 10 μg of protein were loaded into each well, and electrophoresis was carried out at 25 mA for approximately 3 h in running buffer composed of 0.1 M Tris-HCl, 0.1 M tricine and 0.1% (*w*/*v*) SDS. The gels were then fixed in a solution of 40% (*v*/*v*) methanol and 10% (*v*/*v*) acetic acid, stained using 0.025% (*w*/*v*) Coomassie Blue G-250 in 10% acetic acid and destaining in 10% (*v*/*v*) acetic acid. Protein concentrations were determined using a bicinchoninic acid assay (BCA, Pierce, Rockford, IL, USA), with bovine serum albumin (BSA) serving as the calibration standard [21].

### 3.5. Antimicrobial Assay

*Candida albicans* was cultured in yeast extract peptone dextrose (YPD) broth (0.5% yeast extract, 1% bacto peptone, 1% d-glucose), *R. solani* in potato dextrose broth (PDB) at 28 °C, *C. michiganensis* subsp*. michiganensis* in nutrient broth (NB) at 28 °C and *S. aureus* in tryptic soy broth (TSB) at 37 °C. The antimicrobial activity of the isolated peptide was tested against three selected microorganisms using microdilution assays. Briefly, microorganisms were collected in mid-log phase and suspended in buffer (low ionic strength buffer; 10 mM sodium phosphate, pH 7.2). Two-fold serial dilutions of the peptide in the same buffer were distributed into sterile 96-well plates (Nunc F96 microtiter plates), after which an aliquot of the cell suspension was added to each well. The cell counts were 2 × 10^5^ CFUs (colony forming units)/mL for bacteria and 1 × 10^5^ CFU/mL for fungi. After incubating the plates for 3 h at 28 °C, 50 μL of each sample were placed on agar plates appropriate for the microorganism being tested. The plates were then incubated for 1 day and the colonies counted. The lowest concentration of peptide that completely inhibited growth was defined as the minimal inhibitory concentration (MIC). Each MIC value was calculated as an average of independent experiments performed in triplicate [22].

Next, 50 μL of each spore suspension were placed in flat-bottomed polystyrene 96-well plates (Nunc, Roskilde, Denmark), and 200 μg of protein (<10 K fraction) dissolved in 50 mM MES buffer were added. After 36–48 h of incubation at 28 °C, the germination and hyphal growth of the spores were microscopically evaluated using an inverted light microscope.

Finally, *C. michiganensis* subsp*. michiganensis* (KCTC 9231) and *S. aureus* (KCTC 1621) were cultured in TSB at 37 °C with agitation, and the antimicrobial activities of the peptide were again determined in microdilution assays. Briefly, bacteria were collected in mid-log phase and suspended in 10 mM sodium phosphate (pH 5.4 and 7.4). Two-fold serial dilutions of the peptide were then distributed into sterile 96-well plates, after which an aliquot of the bacterial cell suspension (5 × 10^5^ CFUs/mL) was added to each well, and the plates were incubated for 4 h at 37 °C. Thereafter, 50 μL of the 20-fold diluted samples were plated onto TSB agar plates. After incubation overnight, the results were assessed [23].

### 3.6. Hemolysis of hRBCs (Human Red Blood Cells)

The hemolytic activities of the peptides were measured in the presence of heparin using hRBCs collected from healthy donors. Fresh hRBCs were rinsed three times in PBS (1.5 mM KH_2_PO_4_, 2.7 mM KCl, 8.1 mM Na_2_HPO_4_, 135 mM NaCl, pH 7.4). Proteins dissolved in PBS were then added to 100 μL of stock hRBCs suspended in PBS (final RBC concentration, 8% v/v), after which the samples were incubated for 60 min at 37 °C with agitation followed by centrifugation for 10 min at 800× *g*. The absorbance (A) of the supernatants was then assessed at 414 nm, with hRBCs in PBS (A_blank_) and 0.1% Triton X-100 (A_triton_) serving as negative and positive controls, respectively. Percent hemolysis was calculated according to the equation:

$$\%\;\text{Hemolysis}=[({\text{A}}_{\text{sample}}-{\text{A}}_{\text{blank}})/({\text{A}}_{\text{triton}}-{\text{A}}_{\text{blank}})]\times 100$$

Each measurement was conducted in triplicate [24].

### 3.7. N-Terminal Amino Acid Sequence Analysis and Mass Spectroscopy of PG-2

The *N*-terminal amino acid sequence of the purified peptide was determined by performing Edman degradation using a pulse liquid automatic sequencer (Applied Biosystems Inc., Foster city, CA, USA, model 473A). BLAST2 homology analysis showed that the amino acid sequence was highly similar to the *C*-terminal portion of potato protease inhibitors, with the best score (43%) obtained for the serine protease inhibitor 1 precursor (PSPI-21-Chain B). The molecular weight, purity and structural characterization of newly isolated AMP (PG-2) were determined using a matrix-assisted laser desorption ionization mass spectrometer (MALDI II, Krator Analytical Inc., Manchester, UK).

## 4. Conclusion

In summary, we have isolated a novel AMP, PG-2, from potato tubers (*Solanum tuberosum* L cv. Gogu Valley). PG-2 appears to possess potent antimicrobial activity against both human or plant pathogens at concentrations ranging from 3.12 to 25 μM, but to exert no hemolytic effects at concentrations up to 40 μM.

## Figures and Tables

**Table 1:** Steps in the purification of PG-2 from potato tubers.

Step	Amount of PG-2 crude proteins (mg/200 g powder)	Yield
1. Protein Extraction Buffer	87.18	43.59
2. Ultrafiltration (under 10 kDa)	11.07	5.55
3. C_18_-HPLC	0.12	0.06

**Table 2:** Minimal inhibitory concentrations (MICs) for PG-2 against bacterial and fungal strains.

Microorganisms	MIC (μM)
*Fungal pathogens*	
*C. albicans*	25
*R. solani*	25
*Bacterial pathogens*	
*S. aureus*	3.12
*C. michiganense* subsp*. michiganensis*	3.12

## References

[1] Osborn W.F., Broekaert F.R., Terras B.P., Cammue R.W. (1995). Plant defensins: Novel antimicrobial peptides as components of the host defense system. Plant Physiol.

[2] Hultmark H.G., Boman D. (1987). Cell-free immunity in insects. Annu. Rev. Microbiol.

[3] Hoffmann J.A. (1995). Innate immunity of insects. Curr. Opin. Immunol.

[4] Park J.-K., Lee R., Gopal C.H., Seo H., Cheong Y. (2012). Isolation and Purification of a Novel Deca-Antifungal Peptide from Potato (*Solanum tuberosum* L cv. Jopung) Against *Candida albicans*. Int. J. Mol. Sci.

[5] Simmaco D., Barra M. (1995). Amphibian skin: A promising resource for antimicrbial peptides. Trends Biotechnol.

[6] Ganz R.I., Lehrer A.K., Lichtenstein T. (1993). Defensins: Antimicrobial and cytotoxic peptides of mammalian cells. Annu. Rev. Immunol.

[7] Sitaram C., Subbalakshmi N. (1998). Mechanism of antimicrobial action of indolicidin. FEMS Microbiol. Lett.

[8] Glazebrook J. (1999). Genes controlling expression of defense responses in *Arabidopsis*. Curr. Opin. Plant Biol.

[9] Hahm J.-Y., Kim S.Y., Lee S.-C., Park S.Y., Shin S.J., Choi Y., Park K.-S. (2007). Purification and antimicrobial activity studies of the *N*-terminal fragment of ubiquitin from human amniotic fluid. Biochim. Biophys. Acta.

[10] Garcia-Olmedo A., Segura M., Moreno F., Madueno A., Molina F. (1999). Snakin-1, a peptide from potato that is active against plant pathogens. Mol. Plant Microb Interact.

[11] Molina M., Berrocal-Lobo A., Segura M., Moreno G., Lopez F., Garcia-Olmedo A. (2002). Snakin-2, an antimicrobial peptide from potato whose gene is locally induced by wounding and responds to pathogen infection. Plant Physiol.

[12] Park M.H., Kim S.C., Park J.Y., Kim S.Y., Lee H.T., Lim H., Cheong K.S., Hahm Y. (2006). Purification and characterization of a heat-stable serine protease inhibitor from the tubers of new potato variety “Golden Valley”. Biochem. Biophys. Res. Commun.

[13] Hahm J.Y., Kim S.C., Park M.H., Kim H.T., Lim Y., Park K.S. (2005). Antimicrobial activity studies on a trypsin-chymotrypsin protease inhibitor obtained from potato. Biochem. Biophys. Res. Commun.

[14] Van Strien L.C., van Loon E.A. (1999). The families of pathogenesis-related proteins, their activities, and comparative analysis of PR-1 type proteins. Physiol. Mol. Plant Pathol.

[15] Garg V.R., Tripathi S., Kumar S.K. (2011). A study on trypsin, *Aspergillus flavus* and *Bacillus* sp. protease inhibitory activity in *Cassia tora* (L.) syn *Senna tora* (L.) Roxb. seed extract. BMC Complement Altern. Med.

[16] Tian S.B., Liu X.C., Wang M.J., Shi Y.Y., Chen Z.H., Hu W.M. (2009). Vegetative storage protein with trypsin inhibitor activity occurs in *Sapindus mukorassi*, a sapindaceae deciduous tree. J. Integr. Plant Biol.

[17] Sano Y.K., Yap Y., Kodama F., Waller K.M., Chung H., Ueda K., Nakamura M., Oldsen H., Yoda Y., Yamaguchi H. (2005). Activation of a novel transcription factor through phosphorylation by WIPK, a wound-induced mitogen-activated protein kinase in tobacco plants. Plant Physiol.

[18] Wu M., Heinrich I.T., Baldwin J. (2011). Two mitogen-activated protein kinase kinases, MKK1 and MEK2, are involved in wounding- and specialist lepidopteran herbivore Manduca sexta-induced responses in *Nicotiana attenuata*. J. Exp. Bot.

[19] Domoney A., Clemente M., Carmen Marín-Manzano E., Jiménez M., Carmen Arques C. (2012). The anti-proliferative effect of TI1B, a major Bowman-Birk isoinhibitor from pea (*Pisum sativum* L.), on HT29 colon cancer cells is mediated through protease inhibition. Br. J. Nutr.

[20] Park J.Y., Kim S.C., Park J.K., Lee S.J., Choi K.S., Hahm Y. (2012). Novel antibacterial activity of β(2)-microglobulin in human amniotic fluid. PLoS One.

[21] Ozols J, Murray P.D. (1990). Amino acid analysis. Guide to Protein Purification.

[22] Park S.C., Park J.Y., Kim J.K., Lee I., Hwang H., Cheong J.W., Nah K.S., Hahm Y. (2009). Antifungal mechanism of a novel antifungal protein from pumpkin rinds against various fungal pathogens. J. Agric. Food Chem.

[23] Park S., Yoo J.Y., Kim S.C., Park Y., do Choi C.H., Seo K.S., Hahm Y. (2011). Effect of acidic pH on antibacterial action of peptide isolated from Korean pen shell (*Atrina pectinata*). J. Pept. Sci.

[24] Park S.C., Park J.Y., Kim C., Jeong S., Yoo K.S., Hahm Y. (2011). A plausible mode of action of pseudin-2, an antimicrobial peptide from *Pseudis paradoxa*. Biochim. Biophys. Acta.

